# Fate of TLR-1/TLR-2 agonist functionalised pDNA nanoparticles upon deposition at the human bronchial epithelium *in vitro*

**DOI:** 10.1186/1477-3155-11-29

**Published:** 2013-08-21

**Authors:** Simon Heuking, Barbara Rothen-Rutishauser, David Olivier Raemy, Peter Gehr, Gerrit Borchard

**Affiliations:** 1 School of Pharmaceutical Sciences Geneva-Lausanne (EPGL), University of Geneva, University of Lausanne, Geneva, Switzerland; 2 Institute of Anatomy, University of Bern, Bern, Switzerland; 3 Vaccine Formulation Laboratory, Department of Biochemistry, University of Lausanne, Lausanne, Switzerland; 4 Adolphe Merkle Institute, University of Fribourg, Fribourg, Switzerland

**Keywords:** Triple cell co-culture, Dendritic cells, Macrophages, Bronchial epithelium, Toll-like receptor agonist, Adjuvant, Chitosan DNA nanoparticles

## Abstract

**Background:**

Plasmid DNA vaccination is a promising approach, but studies in non-human primates and humans failed to achieve protective immunity. To optimise this technology further with focus on pulmonary administration, we developed and evaluated an adjuvant-equipped DNA carrier system based on the biopolymer chitosan. In more detail, the uptake and accompanying immune response of adjuvant *Pam*_*3*_*Cys* (Toll-like receptor-1/2 agonist) decorated chitosan DNA nanoparticles (NP) were explored by using a three-dimensional (3D) cell culture model of the human epithelial barrier. *Pam*_*3*_*Cys* functionalised and non-functionalised chitosan DNA NP were sprayed by a microsprayer onto the surface of 3D cell cultures and uptake of NP by epithelial and immune cells (blood monocyte-derived dendritic cells (MDDC) and macrophages (MDM)) was visualised by confocal laser scanning microscopy. In addition, immune activation by TLR pathway was monitored by analysis of interleukin-8 and tumor necrosis factor-α secretions (ELISA).

**Results:**

At first, a high uptake rate into antigen-presenting cells (MDDC: 16-17%; MDM: 68–75%) was obtained. Although no significant difference in uptake patterns was observed for *Pam*_*3*_*Cys* adjuvant functionalised and non-functionalised DNA NP, ELISA of interleukin-8 and tumor necrosis factor-α demonstrated clearly that *Pam*_*3*_*Cys* functionalisation elicited an overall higher immune response with the ranking of *Pam*_*3*_*Cys* chitosan DNA NP > chitosan DNA NP = DNA unloaded chitosan NP > control (culture medium).

**Conclusions:**

Chitosan-based DNA delivery enables uptake into abluminal MDDC, which are the most immune competent cells in the human lung for the induction of antigen-specific immunity. In addition, *Pam*_*3*_*Cys* adjuvant functionalisation of chitosan DNA NP enhances significantly an environment favoring recruitment of immune cells together with a Th1 associated (cellular) immune response due to elevated IL-8 and TNF-α levels. The latter renders this DNA delivery approach attractive for potential DNA vaccination against intracellular pathogens in the lung (e.g., *Mycobacterium tuberculosis* or influenza virus).

## Background

In general, plasmid DNA (pDNA) vaccines consist of a bacterial plasmid vector, which contains the genetic information encoding for a single- or multi-epitope antigenic protein. pDNA vaccines are usually produced in bacteria (e.g., *Escherichia coli*), purified and injected into the host [1]. When compared to gene therapy, vaccination using pDNA is thought to be effective already at relatively low levels of gene expression. Promising results of pDNA immunisation were obtained in preclinical settings [2], however, studies in non-human primates and humans failed to achieve protective immunity [3-5]. Consequently, amelioration strategies of pDNA vaccination were exploited, ranging from plasmid optimisation, co-formulation with adjuvants to changing to a specific route of administration, e.g., pulmonary vaccination.

Nebulisation of vaccines is regarded as a promising route of immunisation [6] owing to several clinical trials with measles vaccines [7-9], one of which is in clinical phase II/III [9]. It is generally assumed that mimicking the natural way of infection by applying vaccines to the respiratory tract represents an auspicious strategy for the prevention of lung infections (e.g., influenza, measles and tuberculosis).

Further advantages are:

i) delivery of vaccines into the respiratory tract elicits the secretion of local antibodies (IgA), which in turn are capable of crossing epithelia and preventing further entrance of pathogens [10];

ii) the particular non-invasive nature of pulmonary antigen delivery circumvents the common use of needles, which is the major cause for unsafe injections especially in developing countries [11];

iii) use of pulmonary dry powder vaccines may circumvent the common imperative of an intact cold chain for vaccine storage [9];

iv) trained medical personnel may not be required for the administration of vaccines by inhalers.

Next to these benefits, aerosol delivery of pDNA vaccines using poly(ethyleneimine) or polymeric chitosan NP elicited in mice superior antigen-specific immune responses compared to intramuscular injection [12-14]. Furthermore, pulmonary DNA vaccination against the vaccinia virus induced immunity also at different mucosal effector sites (gut, vagina) and protected against subsequent virus challenge, whereas intramuscular immunisation did not [14]. Above mentioned studies on pulmonary DNA vaccination used the PennCentury™ microsprayer, which was shown to deliver micrometer sized droplets into the bronchial tract (bronchi and bronchioles) of mice [15]. In addition, the bronchial tract is considered expedient for vaccination purposes due to less local mucociliary clearance in comparison to the upper airways, which gives rise to increased particle retention times from <24 h to several days [16].

In order to investigate the fate of deposited particles and resulting immune responses within the human bronchial tract, a three-dimensional (3D) cell model of the human bronchial epithelial airway barrier was shown to be a versatile tool next to animal and *ex vivo* systems [17,18]. This 3D cell culture model is composed of a monolayer of human bronchial epithelial cells, with the addition of immune cells: human monocyte-derived macrophages (MDM) on the top and of blood monocyte-derived dendritic cells (MDDC) underneath of the epithelial cell monolayer. Generally, MDM and MDDC belong to the group of antigen-presenting cells (APC) together with B lymphocytes. Uptake of antigens into APC represents the very first and mandatory step towards the induction of an antigen-specific immunity. Among all APC dendritic cells (DC) are the most competent immune cells and have to be targeted by the antigen or an *in situ* antigen-producing system (DNA vaccine) for successful antigen-specific immunisation.

In addition to pulmonary (bronchial) DNA vaccination, another strategy to improve the immunogenicity of DNA vaccines is to include highly purified synthetic adjuvants, which are able to activate distinct parts of the immune system. For effective vaccination, the adjuvant should be co-formulated with the antigen within the same delivery vector [19]. Therefore, a final formulation contains 1) the antigen, 2) the adjuvant, stimulating strongly the innate immune system and 3) a particulate delivery system assuring optimal presentation of 1 and 2 to the immune system (especially DC) [19].

In order to address these necessities, we recently synthesised a novel co-polymer, *CTPPC*, based on polymeric chitosan, as a delivery system [20]. To a new chitosan derivative, 6-*0*-carboxymethyl-*N,N,N*-trimethylchitosan (*CTC*), a *Pam*_*3*_*Cys* moiety, an adjuvant activating the innate immune system, was grafted through a poly(ethylene glycol) (PEG) spacer (final co-polymer abbreviated *CTPPC*). Of note, the *Pam*_*3*_*Cys* molecule has a triacylated moiety and belongs to the group of TLR-1/TLR-2 heterodimer agonists whereas the TLR-2 can also form dimers with TLR-6 when activated with diacylated based agonists (such as *Pam*_*2*_*Cys*). In a second step, *Pam*_*3*_*Cys* functionalised nanoparticles (NP) were prepared by complex coacervation of *CTPPC* with pDNA expressing the green fluorescence protein (GFP) [21]. With regard to the adjuvanticity of the new delivery system, we observed that *Pam*_*3*_*Cys* functionalised pGFP NP induced Interleukin-8 (IL-8) secretion from differentiated THP-1 human macrophages, which was increased by 10-fold compared to non-functionalised carriers [21]. Moreover, both innate immune receptors TLR-1 and TLR-2 are highly expressed in the pulmonary macrophages, dendritic and epithelial cells [22,23]. Consequently, we considered the *CTPPC* NP system for pulmonary (bronchial) DNA vaccination and studied its adjuvanticity using a well-established 3D cell model of the human bronchial epithelial airway barrier. In our study, we aerosolised DNA loaded NP by the PennCentury^TM^ microsprayer onto this 3D cell culture model and examined the uptake of NP by epithelial and immune cells (MDDC and MDM). In addition, we quantified changes in immune response due to NP exposure by measuring the secretions of relevant cytokines, IL-8 and TNF-α.

## Results and discussion

### Chitosan-based DNA NP enter MDM, MDDC and epithelial cells

In order to achieve protective immunity against a desired antigen, the antigen has to reach DC as the most immune competent APC. In contrast to lymphocytes, DC have maintained during their evolution many of so-called pattern recognition receptors (PRR) and thus possess the ability of sensing invasion of bacterial and viral pathogens [24,25]. After encounter of such antigens, immature DC become activated, initiate antigen internalisation and transform into a mature state with high T cell stimulating capability. Thereafter, mature DC migrate to local draining lymph nodes and present the antigen to resident T cells [26]. After successful antigen presentation, naïve T cells become activated, migrate back to the site of antigen exposure within the lung and eliminate infected cells.

In that interplay, pulmonary DC were shown to play a crucial role in lung defense [27,28] and to be imperative for the maintenance of T cell activity as well as for a constant stimulation of T cells, even after their migration to infected lung tissues [29].

However, in view of pulmonary vaccination, human DC are difficult to study, because they are sparse and make up only a small percentage of the pulmonary cell population [30]. In order to investigate the uptake of particulate matter into pulmonary or more precisely bronchial DC, as well as to follow their interplay with macrophages and bronchial epithelial cells *in vitro*, a 3D cell culture model was developed over recent years consisting of bronchial epithelial cells combined with MDM and MDDC [17]. In our study, three different categories of chitosan-based pDNA NP were included for exposition onto this 3D cell culture: 1) empty *CTC* NP, 2) pGFP-loaded *CTC* NP and 3) pGFP-loaded *CTPPC* NP. This experimental design allowed studying the influence of pGFP and TLR-1/2 agonist components. At first, NP were analysed for their physico-chemical properties (Table 1). Similar properties were already reported in our previous publication [31]. It has to be mentioned that these pGFP formulations had a somewhat polydisperse size distribution (PDI around 0.3), which was already noticed for other chitosan pDNA formulations [31,32]. Reducing PDI of *CTC* and *CTPPC* pDNA NP could be achieved in the future using additional agents like poly-γ-glutamic acid [32] or pentasodium tripolyphosphate [33]. For the administration of NP suspensions, we sprayed these onto described cells (at an air-liquid interface) by using the PennCentury™ microsprayer device, which is commonly used for intratracheal administration in mice [15]. This microsprayer creates a fine plume of aerosolised liquid and gives therefore a more realistic way of NP administration than the simple addition of NP suspensions into the corresponding cell culture medium. After exposure for 24 h, cells were fixed and stained for F-actin in addition to the labelling of specific surface markers, CD14 for MDM, and CD86 for MDDC.

**Table 1:** Characteristics of chitosan derivatives: molecular weight (MW) and NP: z averaged particle size (Size), ζ potential (ZP), polydispersity index (PDI) and loading efficiency (LE) of chitosan-based DNA preparations used in this study

**Formulation**	**MW**	**Size**	**ZP**	**PDI**^**a**^	**LE**
	**(g/mol)**	**(nm)**^**a**^	**(mV)**^**a**^		**(%)**^**a**^
*CTC* NP	98,330	232.9 ± 16.1	21.3 ± 1.2	0.248 ± 0.038	n/a
pGFP-loaded *CTC* NP	98,330	304.1 ± 13.0	17.5 ± 1.6	0.342 ± 0.041	92.5 ± 2.5
pGFP-loaded *CTPPC* NP	259,000	327.3 ± 16.2	15.8 ± 1.6	0.366 ± 0.053	89.4 ± 3.8

^a^analyses were performed in triplicates; n/a, not applicable.

**Figure 1:**
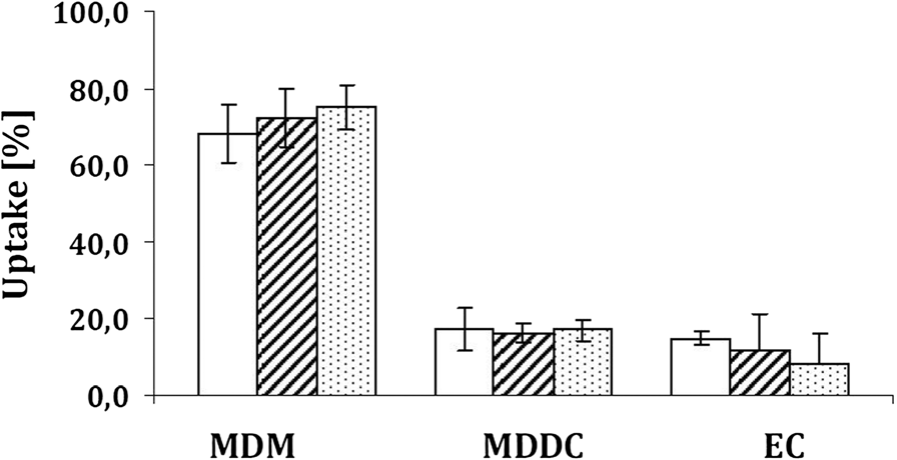
**Uptake of pGFP NP into MDM, MDDC or epithelial cells (EC): unloaded *CTC *NP (white square), *CTC *pGFP NP (black shaded white square) and *CTPPC *pGFP NP (black dotted white square).** Presented data are the mean ± standard error of the mean of three independent experiments. Differences were considered significant for * (*p* < 0.05).

Regarding the overall assessment of NP internalisation into the particular cell types, we were able to assess the percentage of internalised NP with the help of XY- and XZ-projections. As a result, we found that MDM (at the apical side of epithelial cells) phagocytosed the majority of NP applied (68 – 75%). MDDC located at the basolateral side were found to have internalised 16 – 17% of NP, and epithelial cells (EC) ingested a minor percentage of NP of 8 – 15% (see Figure 1). Moreover, the extent of NP uptake was irrespective of pGFP-loading or *Pam*_*3*_*Cys* functionalisation. In previous investigations involving virosomes (0.1 - 0.2 μm in size) and polystyrene particles (0.2 μm and 1.0 μm in size), similar uptake patterns with MDDM > MDDC > epithelial cells were observed [18,28,34]. Regarding the uptake, it can be deduced that the use of chitosan-based NP facilitated mainly (> 85%) transport of the plasmid pGFP into APC (MDM, MDDC), whereas pGFP alone is expected to be degraded upon deposition via extra- and intracellular DNases [35]. In our laboratory, we remarked that the application of pGFP alone did not result in GFP expression in alveolar A549 and bronchial HBE cells, whereas the co-polymer *CTPPC* mediated GFP transfection in both cell lines as evidenced by fluorescence microscopy (*unpublished observations*).

In general, complexation with positively charged polymers shields pDNA from enzymatic degradation by endo- and exonucleases through the formation of polyelectrolyte complexes (PEC). In previous studies we found that complexation with the chitosan polymer protected the plasmid pGFP against the enzyme DNase I, which was in contrast to the complete degradation of pGFP after the same time of enzyme challenge [21]. Although pGFP alone was not studied as a control in this 3D cell culture system, it appears that the use of *CTC* and *CTPPC* for pDNA delivery is favourable due to protection against enzymatic degradation as mentioned above, and most likely higher uptake rates into APC *in vitro*.

Considering different functions of each cell type in this triple *in vitro* model, MDM constitute the first line of defence and are constantly exposed to the entry of antigenic materials into the human lung. MDM also have the ability to inhibit virus growth and to eliminate infected cells. Moreover, activated macrophages can produce antiviral factors (e.g., TNF-α or IFN-α/β) and chemokines (e.g., IL-8), which are able to activate additional cell types in the fight against infections [36]. Although MDM possess only a minor antigen-presenting capacity, a direct cross-talk by exchanging NP with MDDC was proposed [24,37].

**Figure 2:**
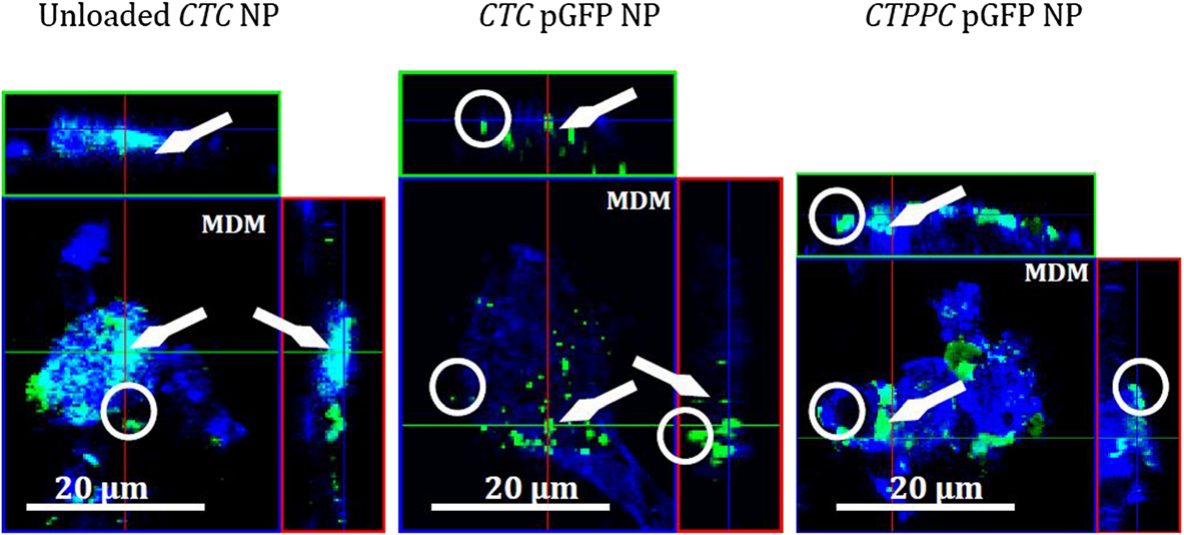
**CLSM micrographs of phagocytosed NP (green) by MDM (blue).** The pictures represent single optical sections; the red line indicates the optical section of the YZ projections (right image) and the green line the XZ projections (upper image). XY and XZ-projections clearly indicate that NP are mainly taken up by MDM (arrow). Extracellular NP are encircled. The horizontal bar represents 20 μm.

**Figure 3:**
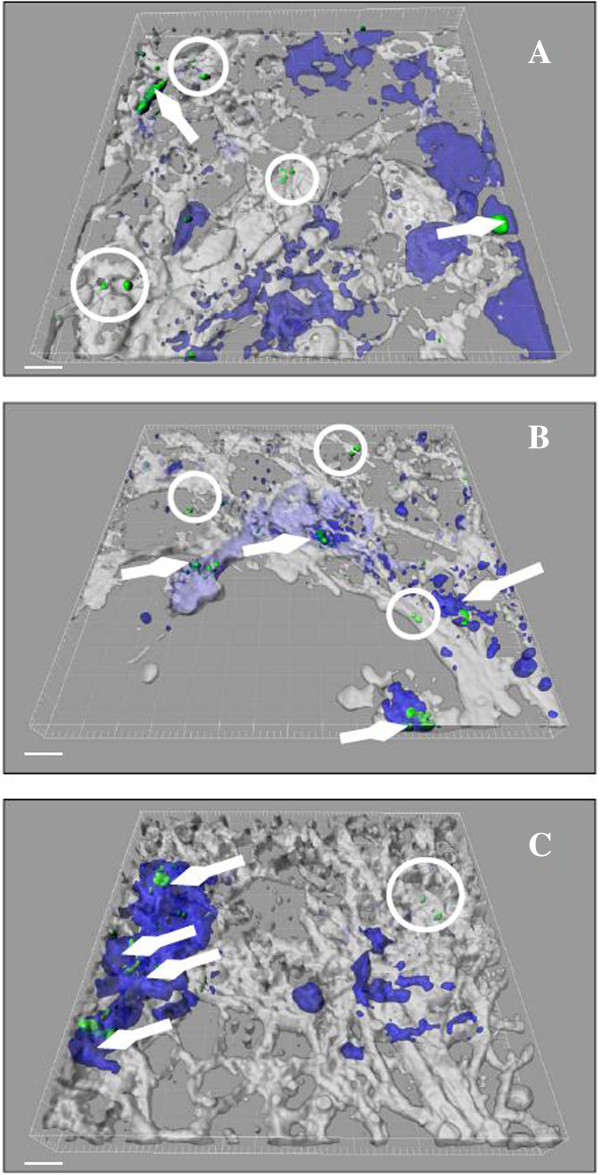
**Visualisation of NP uptake in the apical part of the triple cell culture model (actin, white; macrophages, blue; nanoparticles, green).** The pictures represent 3D renderings of the complete data set and the actin as well as the macrophage labeling was made transparent to show intracellular nanoparticles. **A)** unloaded *CTC* NP, **B)***CTC* pGFP NP and **C)***CTPPC* pGFP NP. Arrows indicate internalised NP into MDM; extracellular NP are encircled. White bar represents a 20 μm scale.

**Figure 4:**
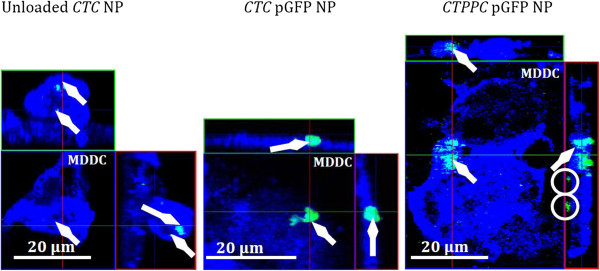
**CLSM micrographs of phagocytosed NP (green) by MDDC (blue).** The pictures represent single optical sections; the red line indicates the optical section of the YZ projections (right image) and the green line the XZ projections (upper image). XY and XZ-projections clearly indicate that NP are internalised by MDDC (arrow). Extracellular NP are encircled. Horizontal bar represents 20 μm.

**Figure 5:**
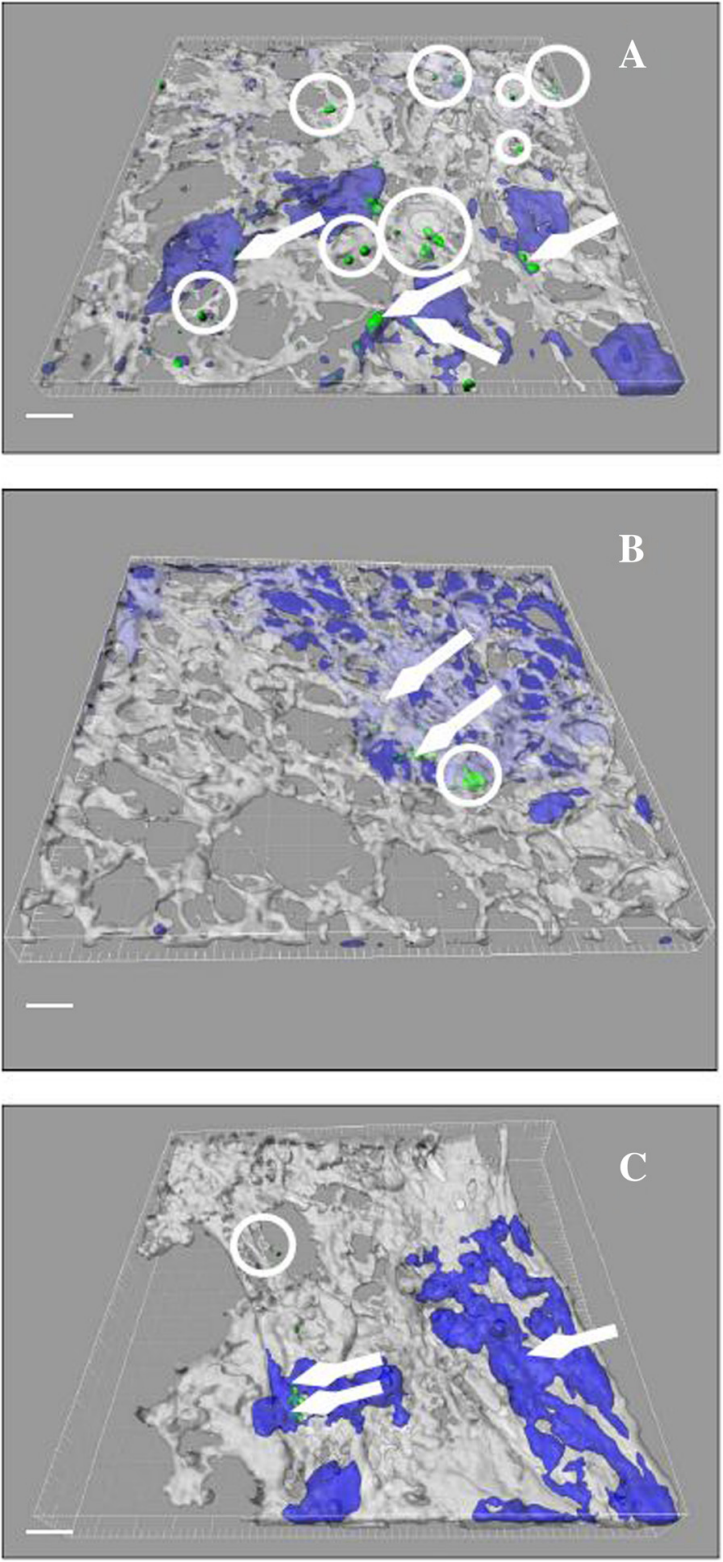
**Visualisation of different NP in the basal compartment of triple cell culture model (actin, white; dendritic cells, blue; nanoparticles, green).** The pictures represent 3D renderings of the complete data set and the actin as well as the dendritic cell labeling was made transparent to show intracellular nanoparticles. **A)** unloaded *CTC* NP, **B)***CTC* pGFP NP and **C)***CTPPC* pGFP NP. Arrow marks internalised NP into MDDC; extracellular NP are encircled. White bar represents a 20 μm scale.

In line with our study, CLSM images of human CD14-positive MDM demonstrated that a pronounced phagocytosis of NP took place (arrow, Figure 2). XY- and XZ-projections also allowed for the recognition of non-phagocytosed NP for comparative purposes (encircled, Figure 2). Furthermore, we reconstructed the apical part of the triple cell co-culture by imaging software, where NP internalisation was detected in all three experimental groups (Figure 3, arrow). Besides, considering the important function of DC as APC, we investigated the capturing of NP by MDDC (located within and beneath the epithelium). It was suggested that MDDC extend pseudopodia even in the absence of apical particles through the epithelium towards the luminal side [38]. Once particles are deposited, MDDC can rapidly induce internalisation (within minutes) followed by transport to the apical side of the epithelium [38]. In our study, we observed uptake of chitosan-based NP into MDDC after 24 h by CLSM (Figure 4). In addition, software reconstructions of the basal part of the 3D cell co-culture model were performed (Figure 5) and corroborated that finding. From these visualisations it becomes clear that MDDC were capable of taking up all three categories of chitosan-based NP.

Considering the uptake mechanism of particulate matter, Blank et al. [38] proposed different ways of particle transport through the epithelium by MDDC: i) uptake of particles through cellular extensions of MDDC across the epithelium, ii) crossing of MDDC through the entire epithelium followed by particle uptake, iii) particle exchange between MDDC, iv) transfer of particles from MDM to MDDC via cell–cell contacts and v) transfer of particles through interactions of MDM with MDDC located within or at the basolateral side of the epithelium. Although we were not able to reveal the exact way of MDDC migration towards NP followed by NP uptake, we found a certain percentage of NP (16-17%) internalised by MDDC after 24 h of exposure. It is highly possible that during the exposure to NP, MDDC, but also MDM, were stimulated by deposited chitosan-based NP. We therefore analyzed for the secretion of two relevant immune mediators, IL-8 and TNF-α.

### TLR-1/2 agonist functionalisation of chitosan DNA NP augments the immune response

In order to investigate a potentially higher adjuvanticity of *CTPPC* pGFP NP due to the adjuvant functionalisation (TLR-1/2 agonist *Pam*_*3*_*Cys*), we studied the expression of two representative cytokines, IL-8 and TNF-α, in the basal compartment of the triple cell co-culture system. With regard to the first cytokine, IL-8 has a role as an essential regulator for the recruitment of leukocytes and their successive trafficking to the mucosal site of infection (chemotaxis). In terms of vaccination, significantly higher levels of IL-8 from human macrophages and monocytes were correlated to a higher adjuvanticity *in vitro* and *in vivo*[38], which was explained by the finding that for particulate adjuvants local recruitment of innate immune cells manifests a critical step. Two licensed adjuvants in Europe, aluminum salts and MF59™, are known to induce IL-8 from human macrophages *in vitro*, which was brought into correlation with mouse *in vivo* data [39].

**Figure 6:**
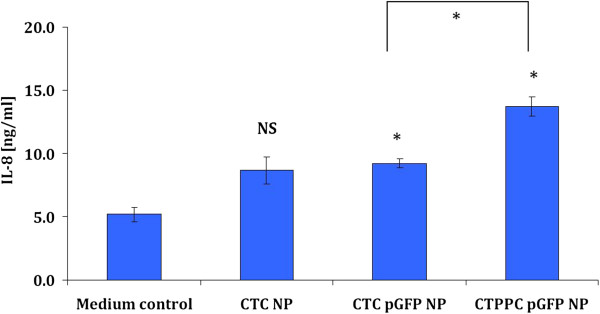
**IL-8 release into the basolateral compartment from triple co-culture cell model due to exposure to unloaded *CTC *NP, *CTC *pGFP NP and *CTPPC *pGFP NP.** Presented data are the mean ± standard error of the mean of three independent experiments. Differences were considered significant for * (*p* < 0.05) in comparison to cells treated with culture medium unless indicated otherwise; NS, not significant.

In our study, we remarked that unloaded *CTC* NP had a weak ability to trigger IL-8 expressions (8.7 ± 1.1 ng/mL), although the difference was not significant (*p* > 0.05) in comparison to control (Figure 6). An IL-8 inducing property of polymeric chitosan was already indicated by Park et al. and might be caused (at least in parts) by complement activation [40]. Secondly, we observed a significant (*p* < 0.05) higher release of IL-8 owing to *Pam*_*3*_*Cys* functionalisation of pGFP NP (*CTC* NP: 9.2 ± 0.4 ng/mL; *CTPPC* NP 13.7 ± 0.8 ng/mL). Spohn et al. [41] and Sadik et al. [42] already described the IL-8 eliciting capacity of *Pam*_*3*_*Cys* on stably TLR-2 transfected HEK cells and differentiated THP-1 macrophages, respectively. The general trend of these IL-8 results goes in line with our previous report [21], in which we noted a 10-fold higher induction of IL-8 from differentiated human-like macrophages (THP-1) by virtue of *Pam*_*3*_*Cys* functionalisation. Next to the chemokine IL-8 and in order to assess the quality of immune responses, we analysed for TNF-α secretions from the 3D cell culture system. TNF-α is an important pro-inflammatory cytokine in host defense and is produced from a variety of immune cells, such as macrophages, T lymphocytes and dendritic cells. TNF-α is associated with a bias towards a T helper cell type 1 (Th1) immune response favouring the induction of cellular immunity. Major functions of TNF-α are recruitment of inflammatory cells, cell differentiation and subsequent elimination of intracellular pathogens. Generally, it appears that the expression levels of TNF-α need to be well balanced. On one side, complete blocking of TNF-α with anti-TNF-α antibodies (as treatment for rheumatoid arthritis) was correlated to increased risks of tuberculosis, viral infections and even reactivated latent infections of tuberculosis in several patients [42]. On the other hand, TNF-α overproduction can cause tissue damage, impair DC development and even septic shock [43]. Of note, the exposure of immature DCs to an aluminum adjuvant and MF59™ did not result in secretion of TNF-α, whereas LPS as TLR-4 agonist elicited significant levels of expression [44].

**Figure 7:**
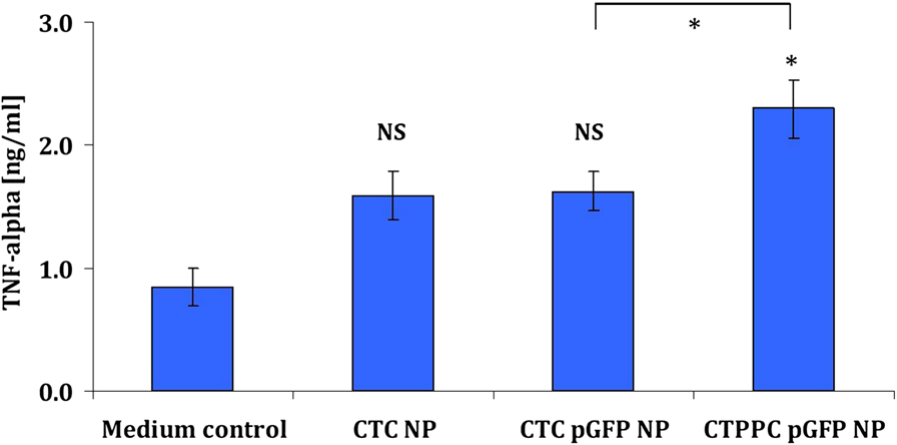
**TNF-α release into the basolateral compartment from triple co-culture cell model due to exposure of unloaded *CTC *NP, *CTC *pGFP NP and *CTPPC *pGFP NP.** Presented data are mean ± standard error of the mean of three independent experiments. Differences were considered significant for * (*p* < 0.05) in comparison to cells treated with culture medium unless indicated otherwise; NS, not significant.

In our study, we found significant secretions of TNF-α being ranked by *CTPPC* pGFP NP > *CTC* pGFP NP = unloaded *CTC* NP > medium control (Figure 7). We remarked that empty *CTC* NP triggered a minor release of TNF-α in comparison to control (not significant). Related to that, Otterlei et al. [45] demonstrated that chitosan (depending on its molecular weight and degree of deacetylation) can induce TNF-α production from human monocytes in a CD14-dependent manner. Interestingly, the additional functionalisation with *Pam*_*3*_*Cys* increased significantly (*p* < 0.05) TNF-α secretion (2.3 ± 0.2 ng/mL), when compared to unmodified *CTC* pGFP NP (1.6 ± 0.2 ng/mL; Figure 7). Schjetne et al. [46] reported that the *Pam*_*3*_*Cys* moiety stimulates CD14-positive human monocytes as well as immature DC via TLR-2 pathway to produce rather high levels of TNF-α (around 1.5 – 2.0 ng/mL), which were in a similar range as those induced by bacterial LPS (as their control) and by *CTTPC* pGFP NP in our study. In addition, more recent immunisation studies in mice support that the distinct *Pam*_*3*_*Cys* moiety stimulates TNF-α expressions in lung tissues [47] and DCs [48].

## Conclusions

The uptake of chitosan-based DNA NP by immune competent MDDC being located at the abluminal side of the human 3D cell culture was demonstrated by using CLSM. ELISA of IL-8 and TNF-α demonstrated clearly that *Pam*_*3*_*Cys* (TLR-1/2 agonist) functionalisation facilitates an overall higher immune response with the ranking of *CTPPC* pGFP NP > *CTC* pGFP NP = unloaded *CTC* NP > medium control. It can be concluded that novel *CTPPC* NP has interesting adjuvant properties due to i) protection against enzymatic degradation; ii) transfection *in vitro*; iii) transport of DNA into the most immune competent APC type, namely DC and iv) increasing the overall immune response (IL-8, TNF-α) being favorable for pulmonary DNA against intracellular pathogens. Currently studies are ongoing in wild type and knockout mice to confirm the potential of the NP systems described here to elicit an immune response on mucosal application.

## Methods

### Triple cell culture

The triple cell culture system was established as previously published [17,38]. Briefly, bronchial 16HBE14o^-^ cells (passage numbers 2.45–2.80) were maintained in MEM 1×, with Earle’s Salts, 25 mM HEPES, without L-Glutamine (Gibco BRL, Switzerland) supplemented with 1% L-Glutamine (LabForce AG, Switzerland), 1% penicillin/streptomycin (Gibco BRL, Switzerland), and 10% fetal calf serum (PAA Laboratories, Lucerna-Chem AG, Switzerland) on transparent BD Falcon cell culture inserts (pores with 3.0 μm diameter, PET membranes for 6-well plates; BD Biosciences, Switzerland) treated with fibronectin coating solution containing bovine serum albumin, 0.1 mg/ml (Sigma, Fluka Chemie GmbH, Switzerland) with 1% bovine collagen, Type I (BD Biosciences, Switzerland) and 1% human fibronectin (BD Biosciences, Switzerland) in LHC Basal Medium (Lucerna Chemie AG, Switzerland). The cells were grown for 7 days.

MDM and MDDC were derived from human blood monocytes as previously described [17]. Briefly, peripheral blood monocytes were isolated from buffy coats (Blood Donation Service, Bern, Switzerland) and cultured in the same medium as used for the epithelial cells except for the supplementation of 5% human serum (Blood Donation Service, Bern, Switzerland) instead of 10% fetal calf serum. Regarding the generation of MDDC, monocytes were cultured for 7 days in medium supplemented with 34 ng/ml of IL-4 (Sigma, Fluka Chemie GmbH, Switzerland) and with 50 ng/ml of GM-CSF (R&D Systems, Oxon, UK), whereas MDM were obtained without any additional supplements. After 7 days in mono-cultures MDM were added at the apical and MDDC at the basolateral side of the epithelial monolayer as described in detail by Rothen-Rutishauser et al. [17].

Next, the triple cell co-cultures were kept overnight in medium supplemented with 1% L-glutamine, 1% penicillin/streptomycin, and 5% heat-inactivated (pooled) human serum (designated as full medium) at 37°C in a 5% CO_2_ humidified atmosphere. The following day the medium was removed completely from the apical chamber while 1.2 mL of medium was kept in the basolateral well to feed the cultures from the basal side of the insert. The cells were exposed to air for 24 h at 37°C in a 5% CO_2_ humidified atmosphere as described earlier [38].

### NP preparation and characterization

Synthesis of *CTC* with a degree of carboxymethylation of 23.2% and degree of trimethylation of 33% and subsequent synthesis of *CTPPC* with a degree of grafting of about 2.1% was performed as described earlier based on an endotoxin-free polymeric chitosan (KitoZyme S.A., Herstal, Belgium) [21]. In addition to our previous publication, we analysed the molecular weight of chitosan derivatives by SEC-MALLS. Hereby, measurements were performed using a TOSOH TSK Gel G3000PW_XL_-CP size exclusion column (TOSOH Bioscience, Germany) with 0.2 M sodium acetate/0.3 M acetic acid (pH 4.4) as eluent (0.3 mL/min). A Waters Alliance HPLC system coupled to a differential refractive index (RI) detector (Schambeck, Germany) and a light scattering detector (MiniDawn, Wyatt, USA) was used for sample handling. Pullan standards ranging from 47,000 g/mol to 710,000 g/mol (PSS, Germany) were used for calibration. Next, in order to fluorescently label NP, we selected an Alexa Fluor 488 sulfodichlorophenol dye (A30052, Invitrogen, France). The Alexa Fluor 488 dye was coupled to free amine moieties of *CTC* and *CTPPC* (at 1% molar ratio of sugar units), respectively, according to manufacturer’s recommendation. Emission spectra (excitation at λ = 495 nm) of labeled chitosan derivative were taken using a FluoroMax spectrometer (Spex, Switzerland) and no quenching was observed. Successively, NP were formed according to our published method [21]. Briefly, *CTPPC* (at 3.10 mg/mL, average molecular weight per sugar unit of 290.8 Da, 3.6 μmol/mL -N^+^(CH_3_)_3_, N) or *CTC* (at 2.21 mg/mL, average molecular weight per sugar unit of 207.1 Da, 3.6 μmol/mL -N^+^(CH_3_)_3_, N) were dissolved in Miniversol water. Separately, the endotoxin-free plasmid pIRES-hrGFP II (abbreviated pGFP; 1 μg of pGFP being equal to 3.1 nmol of phosphate groups, P) was dissolved in 5 mM aqueous sodium sulfate solution at a concentration of 390 μg/mL in order to yield N/P ratios of 3:1. Both solutions were heated for 5 minutes at 55°C. In the following, the polymer containing solution was slowly added (approximately 1 drop per second) to the pGFP solution and vortexed at low speed for 30 seconds. Attention was paid to keep the final volume below or at 400 μL in order to obtain a narrow particle size distribution. Moreover, unloaded *CTC* nanoparticles (control group) were prepared with the help of the cross-linking agent pentasodium tripolyphosphate (TPP) at a molar ratio of 3:1 (positive amines N + (CH_3_)_3_ to TPP molecules) at room temperature via dropwise addition of TPP solution (0.58 mg/mL) to slowly vortexed polymer solution. The final volume was similarly kept at ≤ 400 μL. All particle formulations were kept at room temperature for at least one hour prior to further use. After preparation, hydrodynamic diameters of labelled NP were measured by Photon Correlation Spectroscopy (ZetaSizer 3000 HS, Malvern, Switzerland). For each set of measurements, 400 μL of nanoparticle suspensions were diluted in PBS (pH 7.4) to a total of 1.4 mL. Size distribution data were obtained by the z averaged value of three independent groups of ten measurements. In addition, the zeta potential was measured at least in triplicate via micro-electrophoresis by using an aqueous dip cell (ZetaSizer 3000 HS, Malvern, Switzerland). The loading efficiency of NP was determined by centrifugation of 400 μL of nanoparticle suspension at 16,000 × g for 30 min (Centrifuge 5417C/R, Eppendorf, Germany) and quantification of the unloaded pGFP in the supernatant by PicoGreen assay (Quant-iT PicoGreen, Invitrogen, France) according to the manufacturer’s specifications. Fluorescence was measured with a FluoroMax spectrometer (Spex, Switzerland) at excitation and emission wavelengths of 480 and 522 nm, respectively.

### NP exposure on cell surface

Cytotoxicity for both polymer (IC_50_ > 10 μg/mL) and NP (IC_50_ > 500 μg/mL) had been determined previously in mTHP-1 cells (differentiated monocyte cell line) [21]. Particle concentrations in this study were kept well below cytotoxic concentrations. For exposure, NP suspensions (unloaded *CTC* NP, *CTC* pGFP NP and *CTPPC* pGFP NP, respectively) were extemporaneously prepared before each experiment and were diluted 1:4 in plain cell culture medium in order to yield 4 μg of pGFP in 150 μL medium. After cultivation of cell cultures at the air-liquid interface for 24 h, for each exposure, the respective insert was taken out and placed shortly into another 6-well plate (filled with 1.2 mL of full medium). The diluted NP suspensions (150 μL per 6-well plate) were then sprayed on the apical surface of the triple cell co-culture using a MicroSprayer (model IA-1C, 10" long 0.64-mm tube, PennCentury™, USA). After exposure, the insert was placed back into its original position within the 6-well plate, incubated for another 24 h and then fixed for microscopic analysis. Control experiments were performed with plain cell culture medium. A total of three independent experiments were performed.

### Cell labeling and CLSM

Triple cell co-cultures were fixed and stained after NP exposure and post-incubation as previously reported [17]. Antibodies were diluted in PBS as follows: mouse anti-human CD14 1:20 (Clone UCHM-1, C 7673, Sigma, Switzerland), mouse anti-human CD86 1:20 (Clone HB15e, 36931A, PharMingen, BD Biosciences, Switzerland), goat anti-mouse cyanine 5 1:50 (AP124S, Chemicon, Switzerland), DAPI at 1 μg/mL (Molecular Probes, Switzerland) and rhodamine phalloidin 1:50 (R-415; Molecular Probes, Switzerland). A Zeiss LSM 510 Meta with an inverted Zeiss microscope (Axiovert 200 M, lasers: HeNe 633 nm, HeNe 543 nm, and Ar 488 nm, Diode laser 405 nm; Carl Zeiss AG, Switzerland) was used. Uptake of Alexa488-labeled particles was quantified by using Zeiss LSM Image Examiner (Carl Zeiss AG, Switzerland) on a stack per stack basis [38]. Image processing and visualisation was performed using IMARIS (Bitplane AG, Switzerland), a three-dimensional multi-channel image processing software for CLSM images. For figural representation, LSM and reconstructed images originated from one data set of one randomly selected experiment (out of three independent experiments).

### ELISA of IL-8 and TNF-α

Following 24 h of particle incubation, the cell culture media of the basal chamber of triple cell co-cultures were collected separately and stored at −80°C until further use. After centrifugation, the cytokine tumor necrosis factor-α (TNF-α) and chemokine interleukin-8 (IL-8) concentrations were quantified by a DuoSet ELISA Development kit (DY 210 and DY 208, R&D Systems, UK) according to the manufacturer’s recommendations, except as noted below. The assay was performed in triplicates. An aliquot of 100 μl of the diluted capture antibody (mouse anti-human TNF-α or IL-8, concentration of 4 μg/ml PBS) was incubated overnight in a 96-well immunoassay plate (NUNC, MaxiSorp, Switzerland) at room temperature. Differing from the producer’s protocol, the plate was blocked with PBS supplemented with 1% bovine serum albumin (BSA) and 0.05% NaN_3_ for 1 h at room temperature. After washing with buffer, supernatants from samples and the standards (0–10 ng/mL of recombinant human TNF-α and 0–2 ng/mL of recombinant human IL-8) were pipetted into the wells and incubated at room temperature for 2 h. After washing, the detection antibody (biotinylated goat anti-human TNF-α or IL-8, respectively) diluted in reagent diluent was added.

The plate was covered with an adhesive strip and incubated again for 2 h. After washing horseradish peroxidase-conjugated streptavidin was added to the plates and incubated for 20 min at room temperature in the dark. Finally, the substrate solution (tetramethylbenzidine/H_2_O_2_; DY 999, R&D Systems, UK) was added. After 20 min in darkness, the color development was stopped by adding 50 μL of 1 M H_2_SO_4_ and the plate was put on a shaker (differing from the protocol) for 2 minutes. The absorbance was then read at 450 nm using an ELISA reader (Benchmark Plus Microplate Spectrometer, Switzerland). The concentration of the cytokine was determined by comparing the absorbance of the samples with standard samples using log log regressions.

### Statistics

Data from particle uptake quantifications and ELISA experiments were expressed as the mean ± standard error of the mean and were compared by one-way ANOVA using Origin 7.01 software. Differences were considered significant * at *p* < 0.05.

## Abbreviations

APC: Antigen-presenting cells; BSA: Bovine serum albumin; CTC: *6-0-carboxymethyl-N,N,N-trimethylchitosan*; CTPPC: *Poly (ethylene glycol)-α-amido[N*_*α*_*-palmitoyl-oxy-S-[2,3-bis(palmitoyl-oxy)-(2R)-propyl]-[R-cysteinyl]-ω-amido-graft-6-0-carboxymethyl-N,N,N-trimethylchitosan*; CLSM: Confocal laser scanning microscopy; DC: Dendritic cells; EC: Epithelial cells; pGFP: Plasmid DNA expressing green fluorescence protein; IL-8: Interleukin-8; MDDC: Blood monocyte-derived dendritic cells; MDM: Blood monocyte-derived macrophages; NH2-PEG-Pam3Cys: *ω-amido-[N*_*α*_*-palmitoyl-oxy-S-[2,3-bis(palmitoyl-oxy)-(2R)-propyl]-[R]–cysteinyl]-α-amino poly(ethylene glycol)*; NP: Nanoparticles; PDI: Polydispersity index; pDNA: Plasmid DNA; PRR: Pattern recognition receptors; TLR: Toll-like receptor; TNF-α: Tumor necrosis factor-α; TPP: Pentasodium tripolyphosphate.

## Competing interests

The authors declare that they have no competing interests.

## Authors’ contributions

SH performed the experiments, analysed results and drafted the manuscript. DR, BR were involved in the analysis of results. BR, PG and GB oversaw the experimental work and participated in drafting of the manuscript. All authors have read and approved the manuscript.

## References

[B1] Huygen K Plasmid DNA vaccination Microbes Infect 2005 7 932 938 10.1016/j.micinf.2005.03.010 15878683

[B2] Kutzler MA Weiner DB DNA vaccines: ready for prime time? Nat Rev Genet 2008 9 776 788 10.1038/nrg2432 18781156 PMC4317294

[B3] Wang R Doolan DL Le TP Hedstrom RC Coonan KM Charoenvit Y Jones TR Hobart P Margalith M Ng J Weiss WR Sedegah M De Taisne C Norman JA Hoffman SL Induction of antigen-specific cytotoxic T lymphocytes in humans by a malaria DNA vaccine Science 1998 16 476 480 9774275 10.1126/science.282.5388.476

[B4] Wang R Epstein J Baraceros FM Gorak EJ Charoenvit Y Carucci DJ Hedstrom RC Rahardjo N Gay T Hobart P Stout R Jones TR Richie TL Parker SE Doolan DL Norman J Hoffman SL Induction of CD4(+) T cell-dependent CD8(+) type 1 responses in humans by a malaria DNA vaccine Proc Natl Acad Sci U S A 2001 98 10817 10822 10.1073/pnas.181123498 11526203 PMC58557

[B5] Jaoko W Nakwagala FN Anzala O Manyonyi GO Birungi J Nanvubya A Bashir F Bhatt K Ogutu H Wakasiaka S Matu L Waruingi W Odada J Oyaro M Indangasi J Ndinya-Achola J Konde C Mugisha E Fast P Schmidt C Gilmour J Tarragona T Smith C Barin B Dally L Johnson B Muluubya A Nielsen L Hayes P Boaz M Kaleebu P: **Safety and immunogenicity of recombinant low-dosage HIV-1 A vaccine candidates vectored by plasmid pTHr DNA or modified vaccinia virus Ankara (MVA) in humans in East Africa** Vaccine 2008 26 2788 2795 10.1016/j.vaccine.2008.02.071 18440674

[B6] Bivas-Benita M Ottenhoff TH Junginger HE Borchard G Pulmonary DNA vaccination: concepts, possibilities and perspectives J Control Rel 2005 107 1 29 10.1016/j.jconrel.2005.05.028 PMC7114572 16054263

[B7] Dilraj A Cutts FT De Castro JF Wheeler JG Brown D Roth C Coovadia HM Bennett JV Response to different measles vaccine strains given by aerosol and subcutaneous routes to schoolchildren: a randomised trial Lancet 2000 355 798 803 10.1016/S0140-6736(99)95140-1 10711928

[B8] Bennett JV Fernandez De Castro J Valdespino-Gomez JL De Garcia-Garcia LM Islas-Romero R Echaniz-Aviles G Jimenez-Corona A Sepulveda-Amor J Aerosolized measles and measles-rubella vaccines induce better measles antibody booster responses than injected vaccines: randomized trials in Mexican schoolchildren Bull World Health Organ 2002 80 806 812 12471401 PMC2567652

[B9] Simon JK Levine MM Weniger BG Restrepo AMH Levine MM Mucosal immunisation and needle-free injection devices New generation vaccines 2010 London: Informa Healthcare 405 414

[B10] Brandtzaeg P Induction of secretory immunity and memory at mucosal surfaces Vaccine 2007 25 5467 5484 10.1016/j.vaccine.2006.12.001 17227687

[B11] Miller MA Pisani E The cost of unsafe injections Bull World Health Organ 1999 77 808 811 10593028 PMC2557745

[B12] Bivas-Benita M Van Meijgaarden KE Franken KL Junginger HE Borchard G Ottenhoff TH Geluk A Pulmonary delivery of chitosan-DNA nanoparticles enhances the immunogenicity of a DNA vaccine encoding HLA-a*0201-restricted T-cell epitopes of mycobacterium tuberculosis Vaccine 2004 22 1609 1615 10.1016/j.vaccine.2003.09.044 15068842

[B13] Bivas-Benita M Lin MY Bal SM Van Meijgaarden KE Franken KL Friggen AH Junginger HE Borchard G Klein MR Ottenhoff TH Pulmonary vaccination with DNA encoding mycobacterium tuberculosis latency antigen Rv1733c associated to PLGA-PEI nanoparticles enhances T cell responses in a DNA prime/protein boost vaccination regimen in mice Vaccine 2009 27 4010 4017 10.1016/j.vaccine.2009.04.033 19389445

[B14] Bivas-Benita M Bar L Gillard GO Kaufman DR Simmons NL Hovav AH Letvin NL Efficient generation of mucosal and systemic antigen-specific CD8+ T-cell responses following pulmonary DNA immunisation J Virol 2010 84 5764 5774 10.1128/JVI.02202-09 20335249 PMC2876582

[B15] Bivas-Benita M Zwier R Junginger HE Borchard G Non-invasive pulmonary aerosol delivery in mice by the endotracheal route Eur J Pharm Biopharm 2005 61 214 218 10.1016/j.ejpb.2005.04.009 16039104

[B16] Pulliam B Sung JC Edwards DA Design of nanoparticle-based dry powder pulmonary vaccines Expert Opin Drug Deliv 2007 4 651 663 10.1517/17425247.4.6.651 17970667

[B17] Rothen-Rutishauser BM Kiama SG Gehr P A three-dimensional cellular model of the human respiratory tract to study the interaction with particles Am J Respir Cell Mol Biol 2005 32 281 289 10.1165/rcmb.2004-0187OC 15640437

[B18] Rothen-Rutishauser BM Blank F Mühlfeld C Gehr P In vitro models of the human epithelial airway barrier to study the toxic potential of particulate matter Expert Opin Drug Metab Toxicol 2008 4 1075 1089 10.1517/17425255.4.8.1075 18680442

[B19] O’Hagan DT Singh M Ulmer JB Microparticle-based technology for vaccines Methods 2006 40 10 19 10.1016/j.ymeth.2006.05.017 16997709

[B20] Heuking S Iannitelli A Di Stefano A Borchard G Toll-like receptor-2 agonist functionalised biopolymer for mucosal vaccination Int J Pharm 2009 381 97 105 10.1016/j.ijpharm.2009.03.039 19782879

[B21] Heuking S Adam-Malpel S Sublet E Iannitelli A Stefano A Borchard G Stimulation of human macrophages (THP-1) using toll-like receptor-2 (TLR-2) agonist decorated nanocarriers J Drug Target 2009 17 662 670 10.1080/10611860903106034 19694614

[B22] Muzio M Polentarutti N Bosisio D Prahladan MK Mantovani A Toll-like receptors: a growing family of immune receptors that are differentially expressed and regulated by different leukocytes J Leukoc Biol 2000 67 450 456 10770275 10.1002/jlb.67.4.450

[B23] Nishimura M Naito S Tissue-specific mRNA expression profiles of human toll-like receptors and related genes Biol Pharm Bull 2005 28 886 892 10.1248/bpb.28.886 15863899

[B24] Lambrecht BN Prins JB Hoogsteden HC Lung dendritic cells and host immunity to infection Eur Respir J 2001 18 692 704 11716176

[B25] Foged C Sundblad A Hovgaard L Targeting vaccines to dendritic cells Pharm Res 2002 19 229 238 10.1023/A:1014474414097 11934227

[B26] Randall TD Pulmonary dendritic cells: thinking globally, acting locally J Exp Med 2010 207 451 454 10.1084/jem.20100059 20212067 PMC2839142

[B27] Nicod LP Pulmonary defence mechanisms Respiration 1999 66 2 11 10.1159/000029329 9973683

[B28] Blank F Von Garnier C Obregon C Rothen-Rutishauser B Gehr P Nicod L The role of dendritic cells in the lung: what do we know from in vitro models, animal models and human studies? Exp Rev of Resp Med 2008 2 215 233 10.1586/17476348.2.2.215 20477250

[B29] McGill J Van Rooijen N Legge KL Protective influenza-specific CD8 T cell responses require interactions with dendritic cells in the lungs J Exp Med 2008 205 1635 1646 10.1084/jem.20080314 18591411 PMC2442641

[B30] Holt PG Pulmonary dendritic cells in local immunity to inert and pathogenic antigens in the respiratory tract Proc Am Thorac Soc 2005 2 116 120 10.1513/pats.200502-017AW 16113478

[B31] Erbacher P Zou S Bettinger T Steffan AM Remy JS Chitosan-based vector/DNA complexes for gene delivery: biophysical characteristics and transfection ability Pharm Res 1998 15 1332 1339 10.1023/A:1011981000671 9755882

[B32] Lee PW Peng SF Su CJ Mi FL Chen HL Wei MC Lin HJ Sung HW The use of biodegradable polymeric nanoparticles in combination with a low-pressure gene gun for Transdermal DNA delivery Biomaterials 2008 29 742 751 10.1016/j.biomaterials.2007.10.034 18001831

[B33] Csaba N Köping-Höggård M Alonso MJ Ionically crosslinked chitosan/tripolyphosphate nanoparticles for oligonucleotide and plasmid DNA delivery Int J Pharm 2009 382 205 214 10.1016/j.ijpharm.2009.07.028 19660537

[B34] Hofer U Lehmann AD Waelti E Amacker M Gehr P Rothen-Rutishauser B Virosomes can enter cells by non-phagocytic mechanisms J Liposome Res 2009 19 301 309 10.3109/08982100902911612 19863165

[B35] Borchard G Chitosans for gene delivery Adv Drug Deliv Rev 2001 52 145 150 10.1016/S0169-409X(01)00198-3 11718938

[B36] Murphy EA Davis JM Brown AS Carmichael MD Van Rooijen N Ghaffar A Mayer EP Role of lung macrophages on susceptibility to respiratory infection following short-term moderate exercise training Am J Physiol Regul Integr Comp Physiol 2004 287 1354 1358 10.1152/ajpregu.00274.2004 15308485

[B37] Blank F Wehrli M Lehmann A Baum O Gehr P Von Garnier C Rothen-Rutishauser BM Macrophages and dendritic cells express tight junction proteins and exchange particles in an in vitro model of the human airway wall Immunobiology 2010 216 86 95 20362352 10.1016/j.imbio.2010.02.006

[B38] Blank F Rothen-Rutishauser B Gehr P Dendritic cells and macrophages form a transepithelial network against foreign particulate antigens Am J Respir Cell Mol Biol 2007 36 669 677 10.1165/rcmb.2006-0234OC 17272826

[B39] De Gregorio E D’Oro U Wack A Immunology of TLR-independent vaccine adjuvants Curr Opin Immunol 2009 21 339 345 10.1016/j.coi.2009.05.003 19493664

[B40] Park CJ Gabrielson NP Pack DW Jamison RD Johnson AJW The effect of chitosan on the migration of neutrophil-like HL60 cells mediated by IL-8 Biomaterials 2009 30 436 444 10.1016/j.biomaterials.2008.09.060 18977028

[B41] Spohn R Buwitt-Beckmann U Brock R Jung G Ulmer AJ Wiesmüller KH Synthetic lipopeptide adjuvants and toll-like receptor 2-structure-activity relationships Vaccine 2004 22 2494 2499 10.1016/j.vaccine.2003.11.074 15193414

[B42] Sadik CD Hunfeld KP Bachmann M Kraiczy P Eberhardt W Brade V Pfeilschifter J Mühl H Systematic analysis highlights the key role of TLR2/NF-κB/MAP kinase signaling for IL-8 induction by macrophage-like THP-1 cells under influence of borrelia burgdorferi lysates Int J Biochem Cell Biol 2008 40 2508 2521 10.1016/j.biocel.2008.04.014 18571457

[B43] Jacobs M Togbe D Fremond C Samarina A Allie N Botha T Carlos D Parida SK Grivennikov S Nedospasov S Monteiro A Le Bert M Quesniaux V Ryffel B Tumor necrosis factor is critical to control tuberculosis infection Microbes Infect 2007 9 623 628 10.1016/j.micinf.2007.02.002 17409008

[B44] Seubert A Monaci E Pizza M O’Hagan DT Wack A The adjuvants aluminum hydroxide and MF59 induce Monocyte and granulocyte chemoattractants and enhance Monocyte differentiation toward dendritic cells J Immunol 2008 180 5402 5412 18390722 10.4049/jimmunol.180.8.5402

[B45] Otterlei M Vårum KM Ryan L Espevik T Characterization of binding and TNF-alpha-inducing ability of chitosans on monocytes: the involvement of CD14 Vaccine 1994 12 825 832 10.1016/0264-410X(94)90292-5 7526573

[B46] Schjetne KW Thompson KM Nilsen N Flo TH Fleckenstein B Iversen JG Espevik T Bogen B Cutting edge: link between innate and adaptive immunity: toll-like receptor 2 internalizes antigen for presentation to CD4+ T cells and could be an efficient vaccine target J Immunol 2003 171 32 36 12816980 10.4049/jimmunol.171.1.32

[B47] Barrenschee M Lex D Uhlig S Effects of the TLR2 agonists MALP-2 and Pam3Cys in isolated mouse lungs PLoS ONE 2010 5 e13889 10.1371/journal.pone.0013889 21124967 PMC2987752

[B48] Lim SN Kuhn S Hyde E Ronchese F Combined TLR stimulation with Pam3Cys and poly I:C enhances Flt3-ligand dendritic cell activation for tumor immunotherapy J Immunother 2012 35 670 679 10.1097/CJI.0b013e318270e135 23090076

